# Metabolic Syndrome Increases the Risk of Kidney Stone Disease: A Cross-Sectional and Longitudinal Cohort Study

**DOI:** 10.3390/jpm11111154

**Published:** 2021-11-06

**Authors:** Che-Wei Chang, Hung-Lung Ke, Jia-In Lee, Yung-Chin Lee, Jhen-Hao Jhan, Hsun-Shuan Wang, Jung-Tsung Shen, Yao-Hsuan Tsao, Shu-Pin Huang, Jiun-Hung Geng

**Affiliations:** 1 Department of Urology, Kaohsiung Municipal Siaogang Hospital, Kaohsiung 812, Taiwan; freshrogerchang@gmail.com (C.-W.C.); leeyc12345@yahoo.com.tw (Y.-C.L.); ghostdeityj@gmail.com (J.-H.J.); whs524@gmail.com (H.-S.W.); uro.shenjt@gmail.com (J.-T.S.); paranoid289@gmail.com (Y.-H.T.); 2 Department of Urology, Kaohsiung Medical University Hospital, Kaohsiung Medical University, Kaohsiung 80756, Taiwan; hunglungke@yahoo.com.tw (H.-L.K.); shpihu73@gmail.com (S.-P.H.); 3 Department of Urology, School of Medicine, College of Medicine, Kaohsiung Medical University, Kaohsiung 80756, Taiwan; 4 Graduate Institute of Clinical Medicine, College of Medicine, Kaohsiung Medical University, Kaohsiung 80756, Taiwan; 5 Graduate Institute of Medicine, College of Medicine, Kaohsiung Medical University, Kaohsiung 807, Taiwan; 6 Department of Psychiatry, Kaohsiung Medical University Hospital, Kaohsiung Medical University, Kaohsiung 80756, Taiwan; u9400039@gmail.com; 7 Research Center for Environmental Medicine, Kaohsiung Medical University, Kaohsiung 80756, Taiwan

**Keywords:** epidemiologic study, longitudinal study, kidney stone, metabolic syndrome, risk factors, diabetes mellitus, hypertension, dyslipidemia

## Abstract

We aimed to examine the association between metabolic syndrome and the risk of kidney stone development in a large-scale community-based cohort. A total of 121,579 participants enrolled in the Taiwan Biobank were analyzed. They were divided into two groups on the basis of presence of metabolic syndrome. The presence of kidney stone disease was defined by self-reported history of kidney stones. The mean age of participants was 50 years old, and self-reported kidney stones were observed in 3446 (10%) and 4292 (5%) participants with metabolic syndrome and without metabolic syndrome, respectively. Higher prevalence of kidney stone disease was found in participants with metabolic syndrome compared to those without metabolic syndrome (odds ratio (OR), 1.32; 95% confidence interval (95% CI), 1.25 to 1.39). In addition, the risk of incident kidney stone development was analyzed in a longitudinal cohort of 25,263 participants without kidney stones at baseline during a mean follow-up of 47 months. Multivariable Cox regression analysis revealed that the risk for incident kidney stone disease was higher in participants with metabolic syndrome than those without metabolic syndrome (hazard ratio, 1.24; 95% CI, 1.04 to 1.49). Our study suggests that metabolic syndrome does increase the risk of kidney stones.

## 1. Introduction

Kidney stone disease (KSD) is a global health problem with an increasing cumulative incidence in the U.S. and other countries [1]. The overall lifetime risk of KSD in Taiwan is around 10% [2], which is similar to global trends [3]. Kidney stones could lead to ureteral obstruction, resulting in life-threatening conditions such as septic shock. It could also result in chronic kidney diseases [4]. The pathophysiology of stone formation is a multifactorial combination of genetics, dietary, metabolic, and environmental components [5]. Many associated risk factors have been reported in the past few decades. Obesity, diabetes mellitus (DM) and hypertension are linked to increased risk of stone formation. Therefore, it is crucial for us to identify modifiable factors in order to prevent or decrease the risk of renal stone disease.

Metabolic syndrome comprises of hypertension, obesity, hyperlipidemia, and hyperglycemia. The correlation between metabolic syndrome and renal stones has been reported in several cross-sectional studies [6,7]. However, only few longitudinal cohort studies have been reported [8]. We aimed to examine the association between metabolic syndrome and the risk of kidney stone development in a large-scale community-based cohort in Taiwan.

## 2. Materials and Methods

### 2.1. Data Source and Study Population

The data acquisition was from Taiwan Biobank (TWB), a large-scale community-based research database comprised of cancer-free volunteers aged between 30 and 70 years. It enrolled through 29 recruitment centers in Taiwan since 2008. The detailed profile and methods concerning the development of TWB has been described in previous studies [9]. A total of 121,579 participants with adequate baseline were enrolled in the cross-sectional study, as shown in Supplementary Figure S1.

In the longitudinal cohort, participants with known underlying KSD (N = 1946) were excluded. A total of 25,263 participants were examined in the final analysis (Supplementary Figure S1). Participants underwent serial physical examination, biospecimen collection, and questionnaire surveys every 2 to 4 years from 2008 to 2019. Written informed consent was obtained from all participants and all investigations were conducted according to the Declaration of Helsinki. This study was approved by the Institutional Review Board of Kaohsiung Medical University Hospital (KMUHIRB-E(I)-20190398).

### 2.2. Metabolic Syndrome Definition and Assessments

Metabolic syndrome was diagnosed if three or more of the following five traits are present, according to the definition proposed by the Bureau of Health Promotion (Taiwan) in 2006 [10]: central obesity defined as waist circumference ≧90 cm (men) or ≧80 cm (women), blood pressure ≧130/85 mmHg, serum fasting triglyceride level ≧150 mg/dL, serum fasting high-density lipoprotein (HDL) cholesterol level <40 mg/dL (men) or <50 mg/dL (women), and fasting blood sugar ≧100 mg/dL. The related physical examinations and blood tests were completed in all subjects.

### 2.3. Self-Reported KSD

In standardized interviews, participants were asked “Have you ever had KSD?”, “What was the time of diagnosis.” The same questions were repeated during every 2 to 4 years follow-up interviews.

### 2.4. Study Outcome

In the cross-sectional cohort, the association between metabolic syndrome and the prevalence of KSD was evaluated. As for the longitudinal cohort, the primary end point was the development of KSD. As stated above, we had excluded participants with a history of KSD. All subjects in this study had no history of kidney stone at baseline. The association between metabolic syndrome and subsequent KSD was further assessed.

### 2.5. Statistical Analyses

Participants in the present study were stratified into a metabolic syndrome group and a non-metabolic syndrome group. Clinical characteristics were presented as percentages for categorical variables and mean ± standard deviation for continuous variables. The statistical significance of differences among categorical variables was assessed using the Pearson χ^2^ test and among continuous variables was assessed using an independent *t*-test. In the cross-sectional cohort, logistic regression was used to analyze the association between metabolic syndrome and the prevalence of KSD before and after adjusting for potential epidemiologic variables (age, sex, smoke status, alcohol status, marital status, and educational status), and laboratory factors (estimated glomerular filtration rate, serum hemoglobin, serum albumin, and serum uric acid). In the longitudinal cohort, the cumulative event-free survival rates were estimated with Kaplan–Meier analysis and a log-rank test. Event-free survival time was defined as the interval between baseline and primary end point or the last date of follow-up. Participants who died or were lost to follow-up were censored at the date of the last examination. All analyses were carried out using SPSS 20.0 (IBM Corp, Armonk, NY, USA) and R version 3.6.2 (R Foundation for Statistical Computing, Vienna, Austria) and a *p* value of < 0.05 was considered statistically significant.

## 3. Results

### 3.1. Clinical Profiles of the Study Participants

Of the 121,579 included participants, mean ages were 54 ± 10 and 49 ± 11 years in the metabolic syndrome and the non-metabolic syndrome group, respectively. The majority of the subjects (64%) were female and 23% (N = 27,425) had metabolic syndrome (Table 1). The greatest frequency of metabolic syndrome trait regardless of the number of traits presented was increased waist circumference. The second greatest frequency metabolic syndrome trait was hypertension (Table 1). Adults with metabolic syndromes tended to be older with higher blood pressure, serum total cholesterol, triglycerides, blood sugar, uric acid, low density lipoproteins, and have larger waist circumference than those in the non-metabolic syndrome group (Table 1).

### 3.2. Metabolic Syndrome Was Associated with an Increasing Risk of KSD

In univariate binary regression analysis without adjustment, the presence of metabolic syndrome was associated with a 1.79-fold increase of KSD (odds ratio (OR), 1.79; 95% confidence interval (95% CI), 1.70 to 1.88) (Table 2). After adjustment for potential epidemiologic variables, including age, sex, smoke status (never vs. ever), alcohol status (never vs. ever), marital status (yes vs. no), and educational status, the odds decreased to 1.42 (OR, 1.42; 95% CI, 1.35 to 1.49) (Table 2). Similar results were found when adjusted for potentially relevant laboratory factors (estimated glomerular filtration rate, serum hemoglobin, serum albumin, and serum uric acid) (OR, 1.32; 95% CI, 1.25 to 1.39) (Table 2). Furthermore, the presence of more than three metabolic syndrome traits did increase the OR of KSD in a traits-dependent fashion. The OR increased to 2.02 in the subjects with five metabolic syndrome traits (OR, 2.02; 95% CI, 1.76 to 2.33) (Table 2).

### 3.3. The Association between Individual Components of Metabolic Syndrome and KSD

Using the sub-distribution regression model, statistically significant associations of prevalence of KSD and all individual metabolic conditions were observed (Table 3). Among the five individual components of metabolic syndrome, most increased odds of KSD were observed in patients with hypertension (OR, 1.30; 95% CI, 1.24 to 1.37) (Table 3).

### 3.4. Association of Metabolic Syndrome with Incident KSD Development

**Figure 1:**
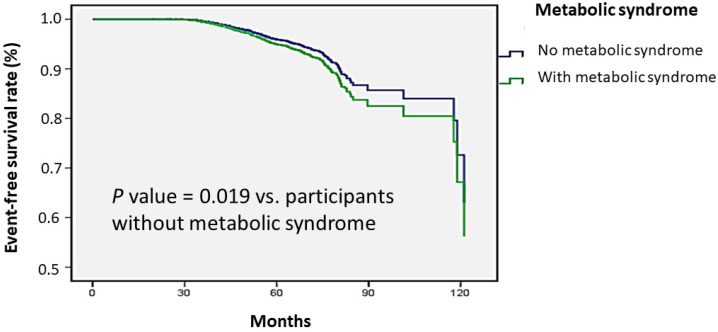
Time to kidney stone disease development was longer in participants without metabolic syndrome than participants with metabolic syndrome. Kaplan–Meier plot of incident kidney stone disease development according to the presence of metabolic syndrome in 25,263 participants with follow-up data.

In the longitudinal cohort, a total of 25,263 participants with no prior KSD were included in the analysis with a mean age of 51 years and 66% were female. Of all participants, 23% had smoking experience and 21% had metabolic syndrome (Supplementary Table S1). A total of 642 participants (3% of the study population) had development of KSD during a mean follow-up duration of 47 months. Multivariable Cox regression analysis revealed that the risk for incident KSD was higher in participants with metabolic syndrome than those without metabolic syndrome (hazard ratio (HR), 1.24; 95% CI, 1.04 to 1.49) (Table 4). Furthermore, subjects with five traits of metabolic syndrome showed the highest risk of developing KSD (HR, 1.54; 95% CI, 0.91 to 2.62) (Table 4). The Kaplan–Meier plot of incident KSD development according to the presence of metabolic syndrome was shown in Figure 1. The time to KSD development was longer in participants without metabolic syndrome than participants with metabolic syndrome (*p* value = 0.019).

## 4. Discussion

In this cross-sectional and longitudinal study of a nationwide population in Taiwan, metabolic syndrome is significantly associated with an increased risk of subsequent KSD after adjustment for covariates. We also noted that the presence of five traits was associated with a more than two-fold increase in odds of KSD, and all components of the metabolic syndrome were associated with a higher prevalence of KSD. To the best of our knowledge, this is the largest longitudinal study on the development of KSD in those with metabolic syndrome, and our results confirmed previous observations that participants with more individual metabolic conditions (hypertension, obesity, DM, and dyslipidemia) had a significantly increased risk of KSD.

Previously, a study of 3872 Korean men without KSD at baseline observed an increased risk of KSD in the metabolic syndrome group compared to the non-metabolic group (HR, 1.771, 95% CI, 1.157 to 2.711) during a mean follow-up duration of 5.77 years [8]. However, it lacked data on women and did not adjust for potential risk factors, such as smoking and drinking. In the present study, we enrolled more than 100,000 participants, and over 20,000 of them had sufficient follow-up. In addition, we collected the information of gender, smoking, drinking, and laboratory factors, which allowed us to adjust for the potential cofounders. The large sample size and detailed information allowed us to examine the cause and effect relationship between metabolic syndrome and the development of KSD during a period of time.

Interestingly, our study showed that among the five individual components of metabolic syndrome, most increased odds of KSD were observed in patients with hypertension. Hypertension is a major risk factor for cardiovascular diseases, and a correlation between KSD and hypertension has been suggested [11]. Two longitudinal studies also found an increased risk of subsequent KSD in hypertensive patients [12,13]. Furthermore, familial hypertensive patients tend to have hypercalciuria and hyperuricosuria, which results in KSD [14].

In line with previous studies, we also found that the presence of increased waist circumference, impaired glucose tolerance, hypertriglyceridemia, and low HDL were associated with an increased risk of KSD [15,16,17,18,19,20]. It is worth noting that our research used waist circumference to represent obesity as opposed to BMI, which could provide an insight into the impact of adiposopathy on KSD. Furthermore, impaired glucose tolerance was used to explore the effects of prediabetes on KSD. These findings could help us with early detection and early control of these diseases to prevent the further development of KSD.

A strength of our study is that we demonstrated a dose-response effect between the traits of metabolic syndrome and the risk of KSD. The odds of KSD were 1.54, 1.81 and 2.02 among subjects with 3, 4, and 5 traits of metabolic syndrome. Similar results were noted in a large Japanese cohort [21]. However, the definitions of metabolic syndrome and individual components in this study did not use ATP III definition. Blood pressure ≧140/90 mm Hg, BMI ≧ 25 kg/m^2^, and fasting plasma glucose level ≧126 mg/dL were used, which could not represent the early status of metabolic syndrome. Furthermore, our study population is bigger than theirs and provides greater power to perform the subgroup analysis.

Several mechanisms have been proposed to explain the association between metabolic syndrome and the development of KSD, including hyperglycemia [19], insulin resistance [22], and vascular dysfunction [23]. Studies have shown that hyperglycemia can increase the secretion of urinary calcium, uric acid, phosphorus, and oxalate, and insulin resistance can lead to a decrease in urine ammonium and PH value, both of which contribute to stone formation [19,22]. Another mechanism proposed to mediate the association between KSD and metabolic syndrome has been vascular dysfunction, which is closely tied to metabolic syndrome. Two studies demonstrated that vascular dysfunction could cause the formation of Randall’s plaques, and initiated the formation of kidney stones [23,24].

The implication of this study is that the incidence of KSD could be reduced by modifying each component in metabolic syndrome. Studies on the Dietary Approaches to Stop Hypertension (DASH) have found that the DASH-style diet could decrease blood pressure, lower oxidative stress induced by acute hyperlipidemia, and reduce the risk of KSD [25,26]. In addition, lifestyle modification, such as diet habit and weight loss could also reduce the risk of KSD as well as other cardiovascular diseases [26].

Our study demonstrated a strong association between metabolic syndrome and KSD. It is also currently the largest longitudinal community-based cohort, with detailed information and regular follow-up. Despite these strengths, there are some limitations in the current study. First of all, self-reported kidney stones were obtained by questionnaires without radiographic verification. Nevertheless, this substitution had been verified in several studies [6,21]. Moreover, Wu et al., reported a moderate concordance between claims records and self-reported renal diseases in Taiwan [27]. Second, we lacked information on diet and daily fluid amount because these factors can easily fluctuate and are difficult to record in detail. Third, we lacked specific information on stone types, but the majority of kidney stones in Taiwan are calcium oxalate, followed by calcium phosphate, and uric acid stones. Magnesium ammonium phosphate stones are rare [28]. Fourth, this study did not include subgroups of children or pregnant women, because this cohort study only included adults and no information about pregnancy status. Fifth, we lacked information regarding performance status, and subjects with poor performance status have a higher risk of developing infection stones [29]. Nevertheless, most participants were 30 to 70 years old, healthy, and cancer-free. Performance status was either 0 or 1.

## 5. Conclusions

Our study demonstrates that metabolic syndrome strongly increases the risk of developing kidney stones as the number of individual traits increases. High blood pressure is the most significant independent component in stone formation. This implies that weight loss, dietary habits, and blood pressure control may reduce the risk of kidney stone development.

## Figures and Tables

**Table 1:** Clinical profiles of the study participants classified by the presence of metabolic syndrome.

Characteristics	Total (N = 121,579)	Metabolic Syndrome (N = 27,425)	No Metabolic Syndrome (N = 94,154)	*p* Value
**Demographic data**				
Age, y	50 ± 11	54 ± 10	49 ± 11	<0.001
Women, n (%)	77,884 (64)	15,443 (56)	62,441 (66)	<0.001
BMI, kg/m^2^	24.2 ± 3.8	27.3 ± 3.8	23.3 ± 3.3	<0.001
Smoke, ever, n (%)	33,151 (27)	9288 (34)	23,863 (25)	<0.001
Alcohol status, ever, n (%)	10,357 (9)	3294 (12)	7063 (8)	<0.001
Physical activity, yes, n (%)	49,296 (41)	11,181 (41)	38,115 (41)	0.394
Married, yes, n (%)	105,040 (86)	24,732 (90)	80,308 (85)	<0.001
Education status, n (%)				<0.001
≦Elementary	6418 (5)	2472 (9)	3946 (4)	
Middle to High school	44,690 (37)	11,759 (43)	32,931 (35)	
≧College	70,471 (58)	13,194 (48)	57,277 (61)	
Systolic BP, mm Hg	120 ± 19	133 ± 18	117 ± 17	<0.001
Diastolic BP, mm Hg	74 ± 11	81 ± 11	72 ± 11	<0.001
**Individual components of metabolic syndrome**				
Hypertension *, n (%)	42,756 (35)	20,537 (75)	22,219 (24)	<0.001
Impaired glucose tolerance †, n (%)	25,291 (21)	15,449 (56)	9842 (11)	<0.001
Increased waist circumference ‡, n (%)	56,467 (46)	24,037 (88)	32,430 (34)	<0.001
Hypertriglyceridemia §, n (%)	25,440 (21)	17,536 (64)	7904 (8)	<0.001
Low high-density lipoprotein ǁ, n (%)	90,490 (74)	17,934 (65)	13,155 (14)	<0.001
**Laboratory data**				
eGFR, mL/min per 1.73 m^2^	103 ± 24	98 ± 24	105 ± 24	<0.001
Hemoglobin, g/dL	13.8 ± 1.6	14.2 ± 1.6	13.6 ± 1.6	<0.001
Albumin, g/dL	4.5 ± 0.2	4.5 ± 0.2	4.5 ± 0.2	<0.001
Fasting glucose, mg/dL	96 ± 21	109 ± 33	92 ± 13	<0.001
Hemoglobin A1c, %	5.8 ± 0.8	6.3 ± 1.2	5.6 ± 0.6	<0.001
Total cholesterol, mg/dL	196 ± 36	200 ± 39	195 ± 35	0.134
Triglyceride, mg/dL	116 ± 94	195 ± 146	93 ± 53	<0.001
HDL cholesterol, mg/dL	55 ± 13	44 ± 9	58 ± 13	<0.001
LDL cholesterol, mg/dL	121 ± 32	124 ± 34	120 ± 31	0.004
Uric acid, mg/dL	5.4 ± 1.4	6.1 ± 1.5	5.2 ± 1.3	<0.001

BMI = body mass index; BP = blood pressure; eGFR = estimated glomerular filtration rate; IQR = interquartile range; HDL = high-density lipoproteins, LDL = low-density lipoproteins. * Systolic blood pressure ≧130 mm Hg or diastolic blood pressure ≧85 mm Hg. † Fasting glucose level ≧100 mg/dL. ‡ Waist circumference ≧ 90cm for men and ≧ 80cm for women. § Serum triglyceride level ≧ 150 mg/dL. ǁ High-density lipoprotein cholesterol level <40 mg/dL for men and <50 mg/dL for women.

**Table 2:** Odds ratios for self-reported kidney stone disease (n = 121,579).

Variables	No. of KSD Cases/No. of Subjects (%)	Unadjusted Odds Ratio (95% CI)	Model 1 Odds Ratio (95% CI)	Model 2 Odds Ratio (95% CI)
**Metabolic syndrome (yes vs. no)**				
No	4292/94,154 (5)	1.00 (Reference)	1.00 (Reference)	1.00 (Reference)
Yes	3446/27,425 (10)	1.79 (1.70 to 1.88)	1.42 (1.35 to 1.49)	1.32 (1.25 to 1.39)
**No. of metabolic syndrome traits**				
0	1305/34,614 (4)	1.00 (Reference)	1.00 (Reference)	1.00 (Reference)
1	1902/33,530 (6)	1.54 (1.43 to 1.65)	1.27 (1.18 to 1.37)	1.24 (1.15 to 1.33)
2	1955/26,010 (8)	2.07 (1.93 to 2.23)	1.54 (1.43 to 1.66)	1.45 (1.35 to 1.57)
3	1438/16,725 (9)	2.40 (2.22 to 2.59)	1.67 (1.54 to 1.80)	1.54 (1.42 to 1.67)
4	847/8182 (10)	2.95 (2.69 to 3.23)	1.99 (1.82 to 2.19)	1.81 (1.64 to 1.99)
5	291/2518 (12)	3.34 (2.92 to 3.81)	2.25 (1.96 to 2.58)	2.02 (1.76 to 2.33)

CI = confidence interval; KSD = kidney stone disease. Model 1 adjusts for potential epidemiologic variables, including age, sex, smoke status, alcohol status, marital status, and educational status. Model 2 adds laboratory factors, including estimated glomerular filtration rate, serum hemoglobin, serum albumin, and serum uric acid to Model 1.

**Table 3:** Odds ratios for self-reported kidney stone disease by individual components of metabolic syndrome (n = 121,579).

Individual Component of Metabolic Syndrome	Unadjusted Odds Ratio (95% CI)	Model 1 Odds Ratio (95% CI)	Model 2 Odds Ratio (95% CI)	Model 3 Odds Ratio (95% CI)
Hypertension *	2.15 (2.06 to 2.26)	1.44 (1.37 to 1.51)	1.37 (1.30 to 1.44)	1.30 (1.24 to 1.37)
Impaired glucose tolerance †	1.79 (1.70 to 1.88)	1.24 (1.17 to 1.30)	1.19 (1.13 to 1.26)	1.11 (1.06 to 1.18)
Increased waist circumference ‡	1.27 (1.21 to 1.33)	1.34 (1.28 to 1.41)	1.26 (1.20 to 1.32)	1.16 (1.10 to 1.22)
Hypertriglyceridemia §	1.65 (1.57 to 1.74)	1.32 (1.25 to 1.39)	1.22 (1.15 to 1.28)	1.12 (1.05 to 1.18)
Low high-density lipoprotein ǁ	1.15 (1.09 to 1.21)	1.22 (1.16 to 1.28)	1.16 (1.10 to 1.23)	1.06 (1.00 to 1.12)

CI = confidence interval. * Systolic blood pressure ≧ 130 mm Hg or diastolic blood pressure ≧85 mm Hg. † Fasting glucose level ≧100 mg/dL. ‡ Waist circumference ≧90 cm for men and ≧80 cm for women. § Serum triglyceride level ≧150 mg/dL. ǁ High-density lipoprotein cholesterol level <40 mg/dL for men and <50 mg/dL for women. Model 1 adjusts for potential epidemiologic variables, including age, sex, smoke status, alcohol status, marital status, and educational status. Model 2 adds laboratory factors, including estimated glomerular filtration rate, serum hemoglobin, serum albumin, and serum uric acid to Model 1. Model 3 adds individual components to Model 2.

**Table 4:** Relative risk for incident kidney stone according to the presence of metabolic syndrome.

Variables	No. of incidental KSD Cases/No. of Subjects (%)	Adjusted Hazard Ratio (95% CI)	*p* Value
**Metabolic syndrome (yes vs. no)**			
No	460/19,948 (2.3)	1.00 (Reference)	-
Yes	182/5315 (3.4)	1.24 (1.04 to 1.49)	0.019
**No. of metabolic syndrome traits**			
0	140/7115 (2)	1.00 (Reference)	-
1	146/7038 (2)	0.94 (0.74 to 1.19)	0.610
2	174/5335 (3)	1.40 (1.11 to 1.77)	0.005
3	119/3225 (4)	1.42 (1.09 to 1.84)	0.008
4	47/1516 (3)	1.30 (0.92 to 1.84)	0.140
5	16/392 (4)	1.54 (0.91 to 2.62)	0.111

CI = confidence interval; KSD = kidney stone disease. Multivariable model: adjustment for age, sex, smoke status, alcohol status, marital status, educational status, estimated glomerular filtration rate, serum hemoglobin, serum albumin, and serum uric acid.

## Data Availability

Restrictions apply to the availability of these data. Data were obtained from Taiwan Biobank and are available with the permission of Taiwan Biobank.

## Supplementary Materials

The following are available online at https://www.mdpi.com/article/10.3390/jpm11111154/s1, Figure S1: Study participants were classified by the presence of metabolic syndrome, Table S1: Clinical characteristics of the longitudinal cohort of participants.
