# Supplementary material for: Metabolic Syndrome Increases the Risk of Kidney Stone Disease: A Cross-Sectional and Longitudinal Cohort Study
Source: J Pers Med. 2021 Nov 6;11(11):1154. doi: 10.3390/jpm11111154 (PMC8622125; doi:10.3390/jpm11111154)

Supplementary Table S1. Clinical characteristics of the longitudinal cohort of participants (N = 25,263)

Characteristics	Value
Age, year	51±10
Female, n (%)	16,771 (66)
Ever smoking, n (%)	5867 (23)
Alcohol status, ever, n (%)	2068 (8)
Education status, n (%)	
≦Elementary	1870 (8)
Middle to High school	11,226 (44)
≧Collage	12,167 (48)
Systolic BP, mmHg	118±18
Diastolic BP, mmHg	73±11
eGFR, mL/min/1.73 m^2	103±24
Uric acid, mg/dL	5.44±1.41
Hemoglobin, g/dl	13.7±1.5
Albumin, g/dl	4.5±0.2
Metabolic syndrome, yes, n (%)	5315 (21)
Follow-up, months	47±14

Abbreviations are the same as in Table 1.

Supplementary Figure S1. Study participants were classified by the presence of metabolic syndrome

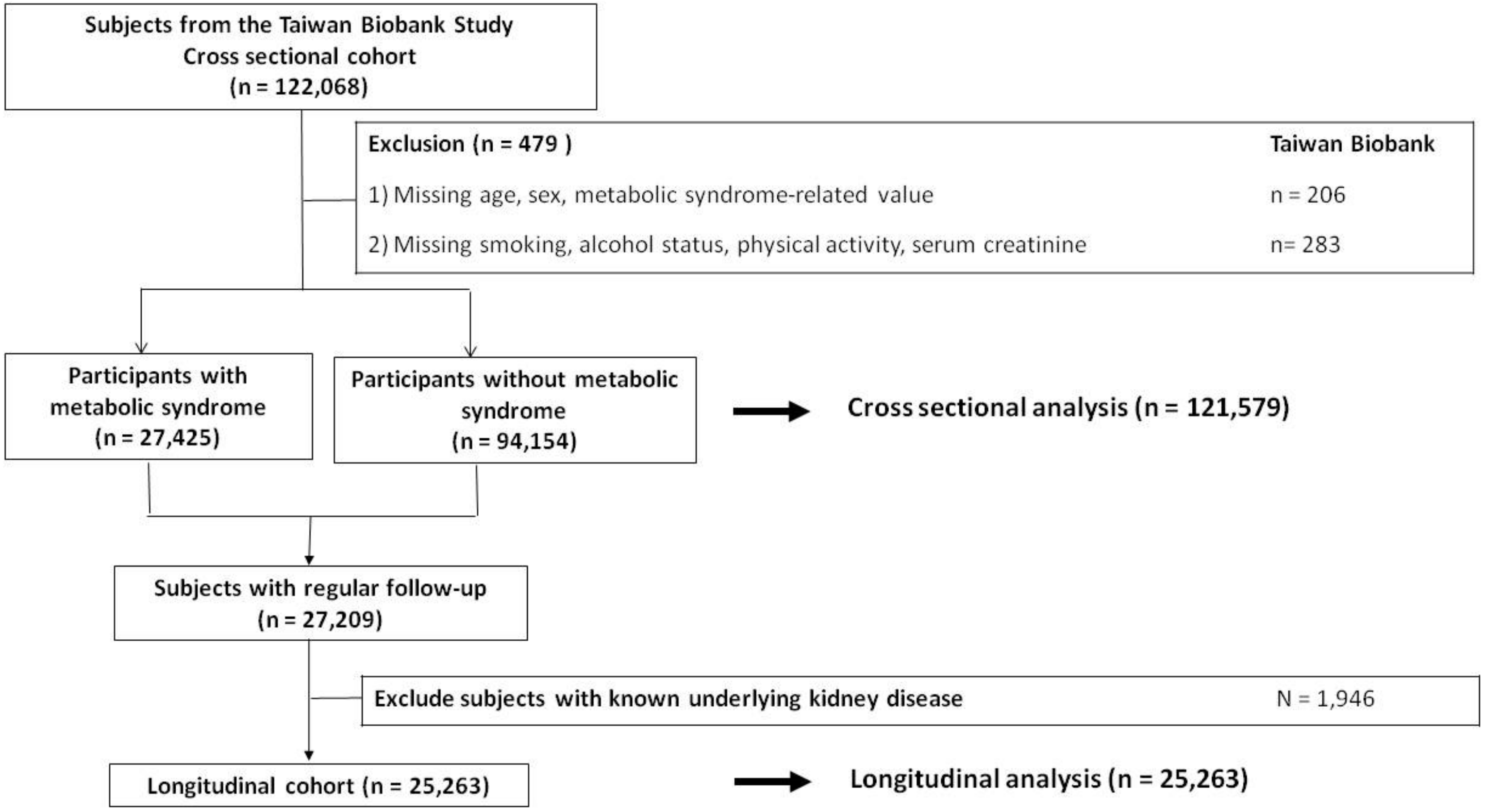

Supplement: Supplementary file 1 [file jpm-11-01154-s001.zip › jpm-1437633-supplementary.pdf]
